# Health profile of centenarians in Portugal: a census-based approach

**DOI:** 10.1186/s12963-016-0083-3

**Published:** 2016-04-12

**Authors:** Oscar Ribeiro, Laetitia Teixeira, Lia Araújo, Constança Paúl

**Affiliations:** Institute of Biomedical Sciences Abel Salazar (UNIFAI and CINTESIS), University of Porto, Rua Jorge Viterbo Ferreira, 228, 4050-313 Porto, Portugal; Polytechnic Institute of Viseu (ESEV and CI&DETS) , Rua Maximiano Aragão, 3504 - 501 Viseu, Portugal

**Keywords:** Centenarians, Health, Cognition, Functional status, Communication, Gender, Census, Longevity, Portugal

## Abstract

**Background:**

The number of centenarians is rapidly increasing in Europe. In Portugal, it has almost tripled over the last 10 years and constitutes one of the fastest-growing segments of the population. This paper aims to describe the health and sociodemographic characteristics of Portuguese centenarians as given in the 2011 census and to identify sex differences.

**Methods:**

All persons living in Portugal mainland and Madeira and Azores islands aged 100 years old at the time of the 2011 census (*N* = 1,526) were considered. Measures include sociodemographic characteristics and perceived difficulties in six functional domains of basic actions (seeing, hearing, walking, cognition, self-care, and communication) as assessed by the Portuguese census official questionnaires.

**Results:**

Most centenarians are women (82.1 %), widowed (82 %), never attended school (51 %), and live in private households (71 %). The majority show major constraints in seeing (67.4 %), hearing (72.3 %), and particularly in their mobility (83.7 % cannot/have great difficulties in walking/climbing stairs and 80.7 % in bathing/dressing). In general, a better outcome was found for reported memory/concentration and understanding, with 39.1 % and 42.5 % presenting no or mild difficulty, respectively. Top-level functioning (no/mild difficulties in all dimensions concurrently) was observed in a minority of cases (5.96 %). Women outnumber men by a ratio of 4.6, and statistically significant differences were found between men and women for all health-related variables, with women presenting a higher percentage of difficulties.

**Conclusion:**

Portuguese centenarians experience great difficulties in sensory domains and basic daily living activities, and to a lesser extent in cognition and communication. The obtained profile, though self-reported, is important in considering the potential of social and family participation of this population regardless of their functional and sensory limitations. Based on the observed differences between men and women, gender-specific and gender-sensitive interventions are recommended in order to acknowledge women’s worse overall condition.

## Background

Although centenarians still represent a small proportion of the total world population, their number is projected to increase rapidly from approximately 441,000 in 2013 to 3.4 million in 2050 and 20.1 million in 2100 [1]. The rise in the number of centenarians has attracted research interest all over the world, particularly for the last two decades, in which several centenarian studies have been conducted in Europe following the examples of long-term centenarian studies conducted in the US and Japan [2].

Collectively, international studies have presented wide-ranging information about centenarians’ sociodemographic characteristics [3], longevity patterns from a psychosocial and health perspective [4], and need for and use of health care services [5, 6], to name a few of the research focus areas. In Portugal, although the number of centenarians has almost tripled over the last 10 years from 589 in 2001 [7] to 1,526 in 2011 [8] and the first population-based study has been recently established, the PT100 Oporto Centenarian Study [9], no large-scale information has been made available on this age group, particularly on their overall health status.

This study aims to present the main sociodemographic characteristics of Portuguese centenarians based on data from last National Census, and to provide a first overview of their elementary health profile in terms of sensory functions (hearing, vision), functional status (walking/climbing stairs and bathing/dressing), cognition (memory/concentration), and communication (understanding/being understood). This set of questions has been developed by the Washington Group on Disability Statistics and is consistent with the International Classification of Functioning, Disability, and Health (ICF) [10]. Sex differences are also investigated.

## Methods

The present study is based on information provided by Statistics Portugal (INE) and collected within the framework of the 2011 census [11, 12]. It considers information about people aged 100 years or more at the moment of census data collection: gender (male or female), marital status (married, single, widowed, or divorced), number of years of education completed (illiterate, 4 years, 6 years, 9 years, 12 years, or higher education), income (pension, family support, properties or business, social support, other), religion (Catholic, other, without religion, or not available), and self-reported disability, which was assessed by a general question about having difficulties doing certain activities due to health problems or aging: difficulty seeing, even with glasses; difficulty hearing, even if using a hearing aid; difficulty walking or climbing steps; difficulty remembering or concentrating; difficulty with self-care such as washing all over or dressing; difficulty communicating (understanding or being understood by others). Each of these questions had three response categories: (1) No difficulty or some difficulty; (2) A lot of difficulty; (3) Cannot do it at all.

Information about type of residence (community or institution) and geographical mobility was also collected. An exploratory analysis was performed in order to characterize Portuguese centenarians. Differences between men and women were examined using chi-square test or Fisher’s exact test (if assumptions of application of chi-square test were not checked). A significance level of *α* = 0.05 was considered.

## Results

In 2011, the number of centenarians in Portugal mainland and Madeira and Azores islands was 1,526, of which 1,253 (82.1 %) were females. Women outnumber men by a ratio of 4.6. The majority of the population was widowed (1,251, 82.0 %), followed by singles (172, 11.3 %), married (86, 5.6 %) and divorced (17, 1.1 %). As for educational level, 940 (61.6 %) centenarians were illiterate, 436 (28.6 %) had completed up to four years of school, and 121 (7.9 %) had completed between 6 and 9 years of school. About half of them (51 %) never attended school (of these, 52.6 % know how to read and write and 47.4 % do not know how to read and write), and only a small minority had higher education (29, 1.9 %).

The great majority (1,084, 71.0 %) lived in private households, and 29 % (442) lived in institutions (e.g., nursing homes). Most centenarians (57 %) were born in the place they currently live (37.5 % in the same town, and 19.5 % in the same council, different town). Monthly income came mostly from their own pension (1,444, 94.6 % of the centenarians), with a minority (4.5 %) relying on their family as the main source of economic support. The majority was Catholic (1,316, 86.2 %).

**Fig 1:**
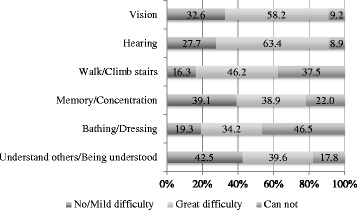
Sensory functions, functionality cognition and communication of Portuguese centenarians

With regard to the centenarians’ sensory functions, functional status, cognition, and communication (Fig. 1), most reported having great difficulties in seeing (58.2 %) and hearing (63.4 %). Understanding others/being understood was the dimension with a higher percentage of individuals presenting no/mild difficulties (649, 42.5 %), and walking/climbing stairs was the dimension with a lower percentage (248, 16.3 %). On the other hand, almost half of the 1,526 centenarians (710, 46.5 %) mentioned being totally unable to take a bath/dress by themselves. Vision and hearing were the dimensions with a lower percentage of total limitation, with 140 (9.2 %) and 136 (8.9 %) centenarians, respectively.

Gender analysis is presented in Table 1. With the exception of income source, all other sociodemographic characteristics of centenarians vary according to gender. Marital status and gender were associated (*p* < 0.001), standing out a high percentage of married males (21.6 %) compared to married females (2.2 %). Analyzing education level, also associated with gender (*p* < 0.001), there was a higher percentage of illiterate females (65.0 %) when compared to males (45.8 %). In both sexes, the majority of centenarians lived in the community; however, an association between sex and type of residence was also observed, with a higher percentage of males living in the community (78.8 % for males and 69.4 % for females, *p* = 0.002). Although the religious profile was similar for males and females, sex and religion were associated (*p* = 0.007), with a slightly higher percentage of males without religion (4.0 % for males and 1.1 % for females).

**Table 1:** Sociodemographic characteristics, sensory functions, cognition, communication, and functionality of Portuguese centenarians by sex

	Sex				*p*
	Female		Male		
Sociodemographic characteristics	n	%	n	%	
Marital status					
Married	27	2.2	59	21.6	<0.001
Single	152	12.1	20	7.3	
Widowed	1061	84.7	190	69.6	
Divorced	13	1.0	4	1.5	
Education (years completed)					
Illiterate	815	65.0	125	45.8	<0.001
4 years	327	26.1	109	39.9	
6 years	33	2.6	9	3.3	
9 years	39	3.1	10	3.7	
12 years	20	1.6	10	3.7	
Higher education	19	1.5	10	3.7	
Residence					
Community	869	69.4	215	78.8	0.002
Institution	384	30.6	58	21.2	
Income source					
Pension	1183	94.4	261	95.6	0.056
Family support	39	3.1	2	0.7	
Properties or business	11	0.9	3	1.1	
Social support	8	0.6	1	0.4	
Other	12	1.0	6	2.2	
Religion					
Catholic	1088	86.8	228	83.5	0.007
Other	19	1.5	6	2.2	
Without religion	13	1.1	11	4.0	
Not available	133	10.6	28	10.3	
Sensory functions, cognition, communication and functionality					
Vision					
No/Mild difficulty	382	30.5	116	42.5	<0.001
Great difficulty	739	59.0	149	54.6	
Can not	132	10.5	8	2.9	
Hearing					
No/Mild difficulty	335	23.7	88	32.2	0.049
Great difficulty	798	63.7	169	61.9	
Can not	120	9.6	16	5.9	
Walk/Climb stairs					
No/Mild difficulty	176	14.0	72	26.4	<0.001
Great difficulty	562	44.9	143	52.4	
Can not	515	41.1	58	21.2	
Memory/Concentration					
No/Mild difficulty	456	36.4	141	51.6	<0.001
Great difficulty	486	38.8	108	39.6	
Can not	311	24.8	24	8.8	
Bathing/Dressing					
No/Mild difficulty	207	16.5	87	31.9	<0.001
Great difficulty	421	33.6	101	37.0	
Can not	625	49.9	85	31.1	
Understand others/Being understood					
No/Mild difficulty	497	39.7	152	55.7	<0.001
Great difficulty	504	40.2	101	37.0	
Can not	252	20.1	20	7.3	

The comparison of sexes according to sensory functions, functional status, cognition, and communication revealed statistically significant differences for all the dimensions under analysis. Overall, females recurrently present greater difficulties in performing activities due to health problem or aging: 10.5 % could not see at all, whereas only 2.9 % of the males were in this situation (*p* < 0.001); being totally unable to hear was reported by 9.6 % of the females and by only 5.9 % of the males (*p* = 0.049); 41.1 % of the females could not walk/climb stairs, a situation reported by 21.2 % of the males (*p* < 0.001); being unable to memorize/concentrate was mentioned by 24.8 % of females and only 8.8 % of males (*p* < 0.001); half of females reported being totally unable to bathe/dress by themselves, whereas this happened in 31.1 % of the male population (*p* < 0.001). In the communication dimension, 20.1 % and 7.3 % of females and males, respectively, mentioned being totally unable to understand others/being understood (*p* < 0.001).

When combined, only 38 (2.49 %) centenarians showed no capacity in all considered dimensions (sensory functions, functionality, cognition, and communication). Of these, most were women (35, representing 2.29 % of the total number of centenarians and 2.79 % of all female centenarians). On the contrary, 91 (5.96 %) centenarians were at the top level of functioning, i.e., presented no/mild difficulties in all considered dimensions concurrently. Most were females (62, 4.06 % of the total centenarians, and 4.95 % of all female centenarians), but within the group of male centenarians there were up to 29 (10.62 %).

## Discussion

This is the first descriptive large-scale health profile of Portuguese centenarians ever conducted. Overall, along with the expected constraints in sensory functions and the presence of great difficulties in basic daily living activities (viz., mobility) and cognition (memory/concentration), a significant proportion of centenarians was found to have no/mild difficulty in understanding others and being understood. Although subjectively measured, these health-related dimensions provide an important basic health profile with implications for service programming. First, it reveals that most centenarians do not generally present a positive outlook and may potentially be in a frail condition; second, it discloses the need to pay attention to this population’s care-provision needs, namely to the maintenance of their capacity to express their wills (ultimately their autonomy) regardless of the reported sensory constraints and functional difficulties.

In being able to understand others/being understood (42.5 %), the capacity of centenarians to express personal resolutions about their own lives according to personal rules and preferences must be acknowledged. This is a crucial aspect of mental health in advanced ages. Considering that centenarians often present high prevalence of diseases and chronic conditions that put them at risk of experiencing limited or restricted participation in society, being able to communicate with no/mild difficulties (expressing wishes, goals, and preferences) ought to be of crucial importance and must therefore be incentivized by care providers, particularly when difficulties in other domains (e.g., functionality, sensory, mobility) may limit independent living or social integration if appropriate accommodations are not made.

In addition to centenarians’ care provision needs (and arguably their health service utilization, though that cannot be inferred from our results), equal attention must be given to their living arrangements, housing conditions, and provision of informal care. Considering that the great majority of centenarians live in private households (71 %), most probably with their immediate family, having a deeper insight into the flows of care provision in multigenerational households is imperative. Encouraging and sustaining family- and community-based care for the elderly is likely to be more cost-effective than residential and nursing care placements, and it is certainly a traditional scenario for Portugal due to the strong tradition of familism that characterizes southern European countries [13], but it raises important questions on the circumstances in which the care is provided. How to care for a very old relative while the caring family members themselves are in advanced age, and how to organize affordable care and medical services that meet the needs of the very old and their families are just two of the currently recognized challenges within centenarian studies [14] that must be taken into account when further analyzing the health circumstances of this population.

As for the second goal of this paper, identifying sex differences, our findings are globally in line with previous studies suggesting that very old men are a minority and tend to present better outcomes than women. Females live longer but suffer a higher level of morbidity, and this has been shown in several studies with the most elderly (e.g., Danish studies [15]) and particularly within centenarian studies from around the world (e.g., China [16], Greece [17]). For instance, in a recent population-based cohort study of centenarians using electronic health records conducted in the UK, authors found that fewer men than women reached the age of 100, and that women had greater multiple morbidity than men, as well as greater likelihood of having multiple geriatric syndromes [18].

In Portugal, we found that centenarian women substantially outnumber men and present an overall worse health status; but we also found that examining the data for women reveals a more vulnerable social condition. Significant differences were found for marital status, educational level, and living arrangements, indicating that on these domains men present a more favorable situation (i.e., having a spouse alive, being non-institutionalized, and having a higher educational level). These findings are easily understood within sociocultural and historical circumstances and can be framed within a gender lens that characterizes this cohort’s life (e.g., men tending to marry younger women, marital status being determined by the mortality rates of spouses, remarriage rates which are more socially acceptable to men). Men and women differ in their life expectancy (shorter for men) and health condition along life (worse for women, on average), and considering the observed differences in the current cohort of centenarians, gender-specific and gender-sensitive approaches to the understanding of health care service needs in very advanced age is to be cautioned.

The feminization of aging is thought to have impact on health outcomes and services, and several authors have argued for a greater focus on the unique needs of women, a gendered approach to policy and intervention development, and the promotion of health across the life span [19]. It is our conviction that such focus ought to include the most elderly population. Particularly in the Portuguese scenario, there is strong evidence of inequity in health against women, and also of the existence of a “gender effect” in health care use [20]. On average Portuguese women report a worse health condition than men, a higher number of disability days, and are more likely to suffer from longstanding illness. The way these conditions link to their socioeconomic status and access to treatment in very old age deserves further attention. Following the trends observed in recent research with centenarians in the Mediterranean that focus on both quantitative and qualitative measures in order to explore perspectives on longevity [21], the study of gender differences and its determinants and consequences should also be conveniently addressed in further studies with this population.

Finally, it is important to highlight a specific sociodemographic aspect of the obtained profile of Portuguese centenarians – the percent of illiteracy/very low educational level. Regardless of the observed differences between men and women on this aspect (more favorable for men), most centenarians never attended school, which is due to sociohistorical reasons (long dictatorship period with scarce access to education). Bearing in mind further research with the Portuguese most elderly population, namely those grouped as “near centenarians,” the use of complex assessment protocols is to be used with caution.

The analysis of the current study relied on a dataset limited to the information available for centenarians, which did not allow for analysis on the trajectory of prevalence and the sex pattern of disability in the most elderly (i.e., beyond 85 years old). This is a weakness of this paper that would be important to overcome in future studies. Such analysis of disability, separately for males and females, should be done to place the analysis of centenarians’ health in perspective.

## Conclusions

This study provides important information about the current sociodemographic profile of Portuguese centenarians and describes their elementary subjective health profile as given by national census data. The high proportion of centenarians presenting great difficulties in sensory domains, basic daily living activities, and to a lesser extent in cognitive (memory/concentration) and communication capacities (understanding others/being understood) reveal the need for more information regarding this population’s specific care needs, their current arrangements of both formal and informal care, and how this may differ from those of younger cohorts of older people. The Washington Group on Disability Statistics’ questions considered in the Portuguese census were designed to provide comparable data cross-nationally and outline a set of domains that were selected using the criteria of simplicity, brevity, universality, and comparability [10]. In being able to capture persons with similar problems across countries, relevant further research will be to compare data on this population (centenarians and most elderly in general) at an international level considering that the WG short set of questions has already been used in a few censuses. Another step will be to analyze the prevalence trajectory of difficulties by age, nationally and cross-nationally. This would provide important information on the population at higher risk for limitations in the ability to fully participate in society due to functional limitations in core domains.

As for the fact that most Portuguese centenarians are living in the community, this finding brings attention to informal caregiving dynamics (and service needs for both care-receivers and caregivers) that might be present in multigenerational households. Finally, the predominance of women among the centenarians and the observed sex differences reaffirm the importance of recognizing gender as a cross-cutting determinant for personal healthy aging trajectories [22]. Differences in health outcomes by sex are common throughout the life-course, but large population-based studies reporting trends in incidence and the health of centenarians are still scarce and should be conducted due to their pivotal role for planning adequate care.
